# Modeling Dipolar Molecules with PCP-SAFT: A Vector Group-Contribution Method

**DOI:** 10.1021/acsomega.4c04867

**Published:** 2024-09-05

**Authors:** Carl Hemprich, Philipp Rehner, Timm Esper, Joachim Gross, Dennis Roskosch, André Bardow

**Affiliations:** † Energy and Process Systems Engineering, Department of Mechanical and Process Engineering, ETH Zurich, Tannenstrasse 3, 8092 Zurich, Switzerland; ‡ Institute of Thermodynamics and Thermal Process Engineering, University of Stuttgart, Stuttgart 70569, Germany

## Abstract

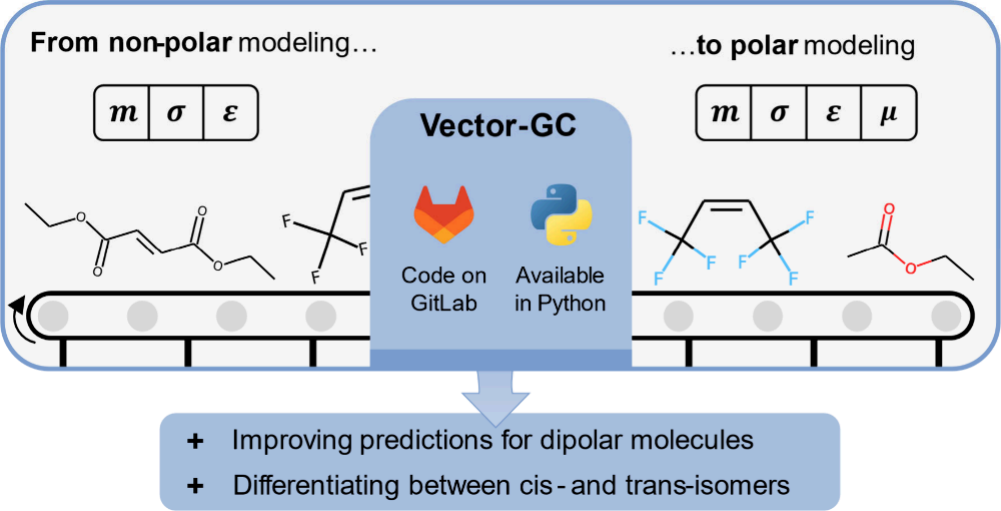

Predicting thermodynamic equilibrium properties is essential to develop chemical and energy conversion processes in the absence of experimental data. For the modeling of thermodynamic properties, statistical associating fluid theory (SAFT)-based equations of state, such as perturbed-chain polar (PCP)-SAFT, have been proven powerful and found broad application. The PCP-SAFT parameters can be predicted by group-contribution (GC) methods. However, their application to the dipole term is substantially limited: current GC methods neglect the dipole term or only allow for a single dipolar group per substance to avoid handling the molecular dipole moment’s symmetry effects. Still, substances with multiple dipolar groups are highly relevant, and their description substantially improves by including the dipole term in SAFT models. To overcome these limitations, this work proposes a vector-addition-based (Vector-)GC method for the dipole term of PCP-SAFT that accounts for molecular symmetry. The Vector-GC employs information on the substance’s molecular 3D structure to predict the molecular dipole moment through a vector addition of bond contributions. Combining the proposed sum rule for dipole moments with established sum rules for the remaining parameters yields a consistent GC method for PCP-SAFT for dipolar substances. The prediction capabilities of the Vector-GC method are analyzed against experimental data for two substance classes: nonassociating oxygenated and halogenated substances. We demonstrate that the Vector-GC method improves vapor pressure and liquid density predictions compared to neglecting the dipole term. Moreover, we show that the Vector-GC method enables differentiation between cis- and trans-isomers. The Vector-GC method, hence, substantially increases the predictive capabilities and applicability domain of GC methods. All parameters are provided as JSON and CSV files, and the Vector-GC method is available through an open-source python package. Additionally, the developed regression framework for GC methods for PCP-SAFT is openly available. The regression framework can be employed to regress the Vector-GC method to other substance classes and is easily adaptable to other sum rules for PCP-SAFT.

## 1 Introduction

Developing novel chemical and energy conversion processes relies on the knowledge of thermodynamic equilibrium properties. Since experimental data is often scarce, reliable property prediction models are required, reducing the need for experimental data and allowing an *in-silico* exploration of the vast chemical space. For this purpose, molecular-based equations of state (EoS) such as the statistical associating fluid theory (SAFT) family have proven to be a powerful tool.^1,2^

In general, SAFT models calculate the residual Helmholtz energy *a*^res^ as a sum of multiple contributions. Since the initial development of SAFT,^3^ several versions have been proposed.^4^ Here, we focus on the Perturbed-Chain Polar (PCP)-SAFT EoS,^5−8^ which is widely applied in research (e.g.,^9−11^) and industry.^12^ PCP-SAFT employs a chain of repulsive spherical segments (hard chain) as a reference fluid. Based on the hard-chain reference, PCP-SAFT defines perturbations to the residual Helmholtz energy that account for molecular interactions. Specifically, the PCP-SAFT EoS describes the residual Helmholtz energy as a sum of the hard-chain reference plus dispersive, associative, and polar contributions.

The hard-chain reference and the dispersive contributions build the core of PCP-SAFT and are sufficient to describe nonpolar and nonassociating substances. Polar and (self-)associating substances usually require the polar and associative contribution terms to explicitly account for the corresponding molecular interactions. Hence, PCP-SAFT requires three to seven substance-specific model parameters to predict molecular properties. The standard parameters *m*, σ, ε represent the number of segments per chain (chain length), segment size, and depth of the potential well. Typically, associating substances are additionally described by the two association parameters ε_AB_ and κ_AB_. Polar substances require the dipole moment μ and the quadrupole moment *Q*.^7,8^ We here follow the approach of Gross and Vrabec^7,8^ and their nomenclature. Still, we would like to highlight the alternative approach by Nguyen Thi et al.^13^ and Nguyen Huynh et al.^14,15^ that introduces the fraction of dipolar and quadrupolar segments in the hard-chain as additional parameters. The approach of Nguyen Thi et al. and Nguyen Huynh et al. is commonly called Polar (P)PC-SAFT to distinguish it from the approach of Gross and Vrabec, which is referred to as PCP-SAFT.

When sufficient experimental data is available, the substance-specific model parameters can be regressed for a given substance. However, experimental data is only available for a limited number of substances, while the chemical space is vast and experimental investigations are resource intensive. To explore the chemical space beyond well-measured substances, parameter prediction methods are highly desirable. In recent decades, model parameters of PCP-SAFT and other SAFT type EoS have been successfully predicted by group-contribution (GC) methods.^16^ The fundamental idea of GC methods is to break down a molecule’s structure into a set of predefined groups, each representing a substructure of the molecule.^17^ GC methods then calculate a molecule’s properties or parameters by summation over group counts and contributions, following so-called sum rules to relate molecular and group parameters. By regressing the group parameters against the experimental data available for a set of well-measured substances, molecular parameters can be predicted for similar, not yet measured substances.

Various GC methods have been developed for predicting PCP-SAFT parameters.^18−27^ A valuable foundation was laid by Vijande et al.,^18^ who proposed sum rules for *m*, σ, and ε by exploiting the linear relationship between *m*, *m* · σ^3^, *m* · ε and the molar mass often observed within an homologous series. Vijande et al. applied the GC method to the homologous series of *n*-alkanes and hydrofluoroethers, represented by a single ether group with one fully fluorinated and one nonfluorinated carbon chain. In subsequent studies, Vijande et al.^19,20^ extended this GC method: First, by accounting for proximity effects in branched substances and by including monofunctional esters.^19^ Then, they considered associative contributions and applied the GC method to monofunctional primary alcohols and amines.^20^ However, Vijande et al. neglected polar contributions.

Employing the sum rules of Vijande et al. for *m*, σ, and ε, Burgess et al.^23^ developed a GC method to predict PCP-SAFT parameters of alkanes, cycloalkanes, and aromatic substances. Following a different approach, Peters et al.^22^ employed Lorentz–Berthelot-inspired sum rules to develop a GC method for *m*, σ, and ε for polymer systems. Moreover, GC methods have been employed indirectly to predict *m*, σ, and ε; for instance, by exploiting physical relations between the parameters and physical properties for which GC methods exist^21,24^ or by building an artificial neural network on the basis of a GC approach.^25^

These existing GC methods for *m*, σ, and ε neglect associative and polar contributions, even though polar substances were investigated.^19,22,25^ However, for the underlying SAFT EoSs, several studies showed that an explicit description of the polar contributions significantly increases their predictive capabilities, e.g., by Gross and Vrabec^8^ for PCP-SAFT, by Nguyen Thi et al.^13^ and Nguyen Huynh et al.^14,15,28^ for PPC-SAFT, or by Cripwell et al.^29^ and Paricaud^30^ for SAFT-VR Mie. In particular, the explicit description of the polar contributions improves the correlation accuracy for pure substances and leads to significantly smaller binary interaction parameters *k*_*ij*_ for the prediction of mixtures. The smaller binary interaction parameters imply greater predictivity, thus also enabling more reliable mixture property prediction in absence of mixture data.^31^

Yet, only very few approaches have been proposed to incorporate polar contributions into a GC method for each PCP-SAFT and PPC-SAFT. Sauer et al.^26^ developed a GC method for PCP-SAFT for a wide range of substance classes, employing the sum rules of Vijande et al.^18^ for *m*, σ, ε and additionally taking associative and polar contributions into account. For this purpose, they defined associating and polar groups with contributions to the association parameters, ε_AB_ and κ_AB_, and the dipole moment μ. Contributions to the quadrupole moment were neglected. These group contributions were simultaneously regressed with the group contributions for *m*, σ, and ε. However, the method of Sauer et al. is limited to monofunctional substances, i.e., those with, at most, a single associating or polar group per substance. Based on the same restriction, Nguyen Thi et al.^13^ and Nguyen Huynh et al.^14,15,28,32^ proposed a GC method for PPC-SAFT incorporating polar contributions for monofunctional substances. Here, additional group contributions are introduced for the fractions of dipolar and quadrupolar segments in the PPC-SAFT model.

The limitation of current GC methods to a single polar group substantially restricts their application range. The reason for imposing the restriction on monofunctional substances is the difficulty associated with defining reasonable sum rules for the dipole moment μ. The fundamental assumption of GC methods is that group contributions are additive. This assumption fails for the dipole moment due to molecular symmetry effects: The molecular dipole moment is a measure for the asymmetrical charge distribution within a molecule, represented as a vectorial quantity by the magnitude and direction of the charge distribution.^33^ When local charges are distributed symmetrically over the molecule, the molecular dipole moment collapses to a vector with zero magnitude. Sum rules for the dipole moment must therefore account for these symmetry effects.

Here, we propose a sum rule for the molecular dipole moment μ that accounts for molecular symmetry. For this purpose, the sum rule employs an estimate of the molecular 3D structure of a substance to predict the dipole moment based on a vector addition of bond contributions. Combining the proposed sum rule for dipole moments with established sum rules for the remaining parameters of PCP-SAFT yields a consistent vector-addition-based (Vector)-GC method for PCP-SAFT parameters. The Vector-GC is parametrized for two classes of nonassociating, dipolar substances: nonassociating oxygenated and halogenated substances. We show that the Vector-GC method outperforms the benchmark case of neglecting polar contributions. Moreover, the Vector-GC enables differentiation between substances’ cis- and trans-isomers, expanding current capabilities of GC methods.

## 2 Vector-GC Method for PCP-SAFT

For the Vector-GC method, we focus on nonassociating, dipolar substances and neglect associating and quadrupolar contributions. Quadrupolar contributions are only significant for few molecules.^31^ The Vector-GC can be extended to associating substances, but since association contributions usually dominate over polar contributions (e.g., shown for alcohols in^34^), we do not expect substantial improvements in the description of associating substances by considering their polarity.

To predict properties of nonassociating, dipolar substances, PCP-SAFT requires 4 parameters: *m* and σ representing the hard-chain reference, ε describing the dispersive contributions, and the dipole moment μ representing polar contributions. We follow an established homo-GC method of Sauer et al.^26^ for the sum rules for *m*, σ, and ε, as described in Section 2.1. In Section 2.2, a novel sum rule for the dipole moment, μ, is introduced, which accounts for molecular symmetries. For this purpose, a simple homo-GC approach is not sufficient, but the Vector-GC method must consider bonds and their three-dimensional orientation in addition to the considered groups. Section 2.3 describes the Vector-GC method’s fragmentation step, which translates a substance’s isomeric SMILES^35^ into groups, bonds, and the bonds’ three-dimensional orientation.

### 2.1 Sum Rules for *m*, σ, and ε

For the nonpolar PCP-SAFT parameters *m*, σ, and ε, we follow the approach of Sauer et al.^26^ by employing the sum rules initially proposed by Vijande et al.:^18^
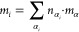
1
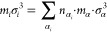
2
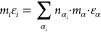
3Here, *m*_*i*_, *σ*_*i*_, and *ε*_*i*_ are the chain length, segment size, and the potential well depth for substance *i*; *n*_*α*_*i*__ is the occurrence of group α in substance *i*, and *m*_*α*_, *σ*_*α*_, ε_α_ are the contributions of group α to the chain length, segment size, and the potential well depth.

### 2.2 Sum Rule for μ

We calculate the *molecular* dipole moment μ based on the concept of *bond* dipole moments, as introduced by Minkin et al.:^36^ Bond dipole moments, *μ*_*β*_, assign a dipole moment contribution to each bond β. A vector sum of the bond dipole moments then yields an estimate of the molecular dipole moment (Equation 4).
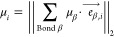
4Here, *μ*_*i*_ is the molecular dipole moment of substance *i*, while *μ*_*β*_ is the bond contribution of bond β, and  is the unit vector of bond β present in substance *i*. The unit vector is defined to always point from the atom with higher electronegativity to the atom with lower electronegativity. The vector sum accounts for molecular symmetries as the unit vectors of bonds of identical type cancel each other out in case of exactly opposite directions.

The unit vectors, , are molecule-specific parameters determined in the fragmentation step, analogously to the groups’ occurrences *n*_*α*_*i*__ in Equations 1–3. In turn, the bond dipole contributions *μ*_*β*_ are transferable parameters that correspond to the group contributions *m*_*α*_, *σ*_*α*_, *ε*_*α*_ and can be regressed to experimental data.

### 2.3 Fragmentation

The Vector-GC method determines the required group occurrences, *n*_*α*_*i*__, and unit vectors, , for a substance *i* from the substance’s SMILES^35^ code. For this purpose, the SMILES code is processed through the open-source python package RDKit^37^ (v2023.03.3).

The group occurrences, *n*_*α*_*i*__, are identified by a substructure search with the RDKit function *GetSubstructMatches*. For the unit vectors, , the RDKit function *GetBonds* first determines the present bonds. Then, an estimate of the substance’s 3D geometry is generated through the RDKit function *EmbedMolecule*, utilizing the distance geometry approach developed by Riniker and Landrum.^38^ This initial 3D geometry estimate is improved by geometry optimization with RDKit’s implementation of the MMFF94 force field, *MMFFOptimizeMolecule*. The resulting 3D coordinates are then used to calculate the unit vectors, , of all present bonds.

The fragmentation step’s execution is rapid, e.g., taking about 14 ms for a molecule with 26 heavy atoms (timed in a jupyter notebook with the python module *timeit* on an AMD EPYC 7F72 workstation CPU).

## 3 Data and Parametrization

This work builds on the parametrization of Sauer et al.^26^ for hydrocarbons, adopting the group definitions and contributions for carbon groups from the Sauer et al. homo-GC method. On this basis, we parametrize the Vector-GC method for oxygenated and halogenated hydrocarbons. Specifically, we consider aliphatic, noncyclic, and nonassociating substances that contain C, H, F, O, Cl, Br, and I atoms.

Section 3.1 defines the groups and bonds considered in this work. The objective function for the regression is presented in Section 3.2. The employed thermophysical property data is introduced in Section 3.3. Finally, Section 3.4 describes the used computational methods and Section 3.5 details the accessibility of the Vector-GC method and its regression framework.

### 3.1 Groups and Bonds

**Figure 1:**
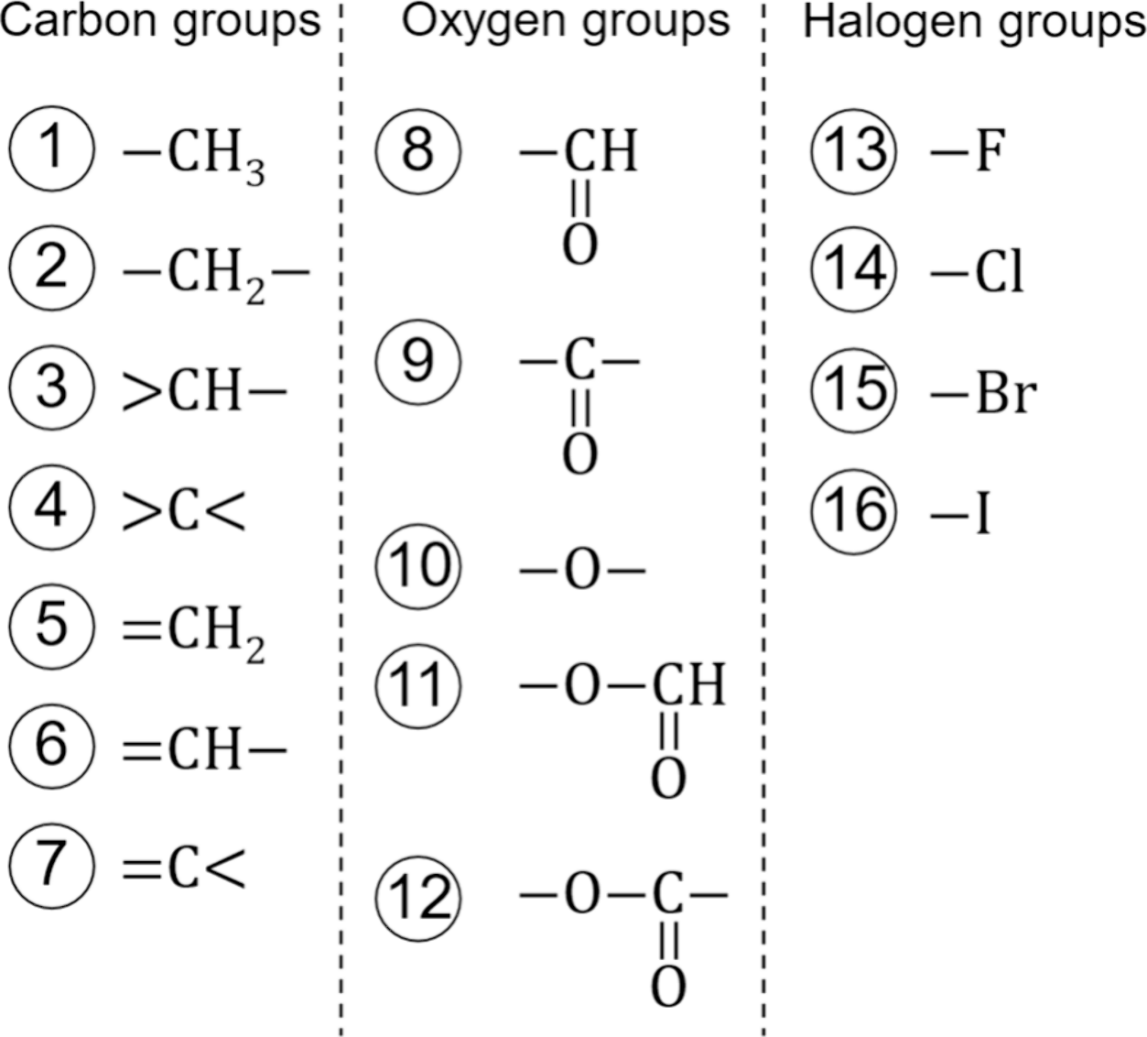
Defined first-order groups categorized in carbon groups (left), oxygen groups (center), and halogen groups (right).

**Figure 2:**
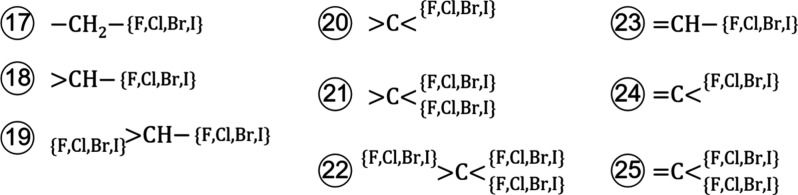
Defined second-order groups for halogenated carbons. The letters in curly brackets indicate adjacent groups, while letters without brackets indicate the second-order group.

For the considered nonassociating oxygenated and halogenated substances, we define 25 groups in total, 16 of which are of first order (Figure 1) and nine of second order (Figure 2).

The seven first-order carbon groups (Figure 1, Groups 1–7) provide the building blocks for alkane and alkene chains. We adopt these group definitions and their contributions (Table 1) from Sauer et al.^26^ With this adoption, the Vector-GC yields the same *m*, σ, ε parameters for alkanes and alkenes as the homo-GC method of Sauer et al. (cf., Section 2.1), ensuring consistency for hydrocarbons.

**Table 1:** Adopted Group Contributions from the Homo-GC Method of Sauer et al.^26^ for the Carbon Groups

Group α	*m*_α_, –	σ_*α*_, Å	ε_α_/k, K
–CH_3_	0.61198	3.7202	229.90
–CH_2_–	0.45606	3.8900	239.01
>CH–	0.14304	4.8597	347.64
>C<	–0.66997	–1.7878	107.68
=CH_2_	0.36939	4.0264	289.49
=CH–	0.56361	3.5519	216.69
=C<	0.86367	3.1815	156.31

Beyond the carbon groups, we define five groups for oxygenated substances (Figure 1, Groups 8–12): an aldehyde (−CH=O), a ketone (>C=O), an ether (−O−), a formate (−O–CH=O), and an ester (−O−(C=O)−) group. The oxygen group definitions are mainly taken from Sauer et al.,^26^ except for the ether group. Sauer et al. defined two ether groups, (CH_3_–O−) and (−CH_2_–O−), differentiating between ether groups connected to a methyl (−CH_3_) and ether groups connected to a methylene group (−CH_2_−). Since this differentiation introduces some ambiguity in the representation of ethers, we define only a single ether group (−O−).

For halogenated substances, we define four first-order groups (Figure 1, Groups 13–16) and nine second-order groups (Figure 2). The first-order groups correspond to individual halogen atoms, fluorine (−F), chlorine (−Cl), bromine (−Br), and iodine (−I). The second-order groups represent halogenated carbons. These second-order carbon groups can correct for differences in the contributions of the adopted first-order carbon groups (Figure 1, Groups 1–7) in the case of halogenation.

The adopted first-order carbon groups were regressed against a set of alkanes and alkenes,^26^ where all open bonds of a group are connected to another carbon group. Hence, we expect the first-order carbon groups to yield well-defined contributions if all or most adjacent groups are carbon groups as well. However, this assumption breaks down for halogenated substances, and the first-order carbon groups represent different parts of the alkane and alkene chains than they were initially regressed to. For instance, the methylene group (−CH_2_−) represents the end of an alkane chain in case of halogenation while representing a middle part otherwise. Additional ambiguity arises for the carbon groups with more than one open bond. For example, the single carbon group (>C<) represents an end, a middle, or a branch part, depending on the degree of halogenation. To account for all cases, we consider the degree of halogenation in the definition of the second-order groups (Figure 2). An alternative approach to defining second-order groups would be to include the first-order carbon groups in the regression. However, this inclusion would lead to a loss of applicability of the first-order carbon groups for other substance classes. Moreover, one could define larger, first-order groups containing halogenated carbons with different halogenation degrees and numbers of open bonds, e.g., −CF_3_ or −CFCl–, as done by Gmehling et al.^39^ This approach, however, lacks flexibility and leads to a high number of defined groups in case all possible combinations should be covered.

**Figure 3:**
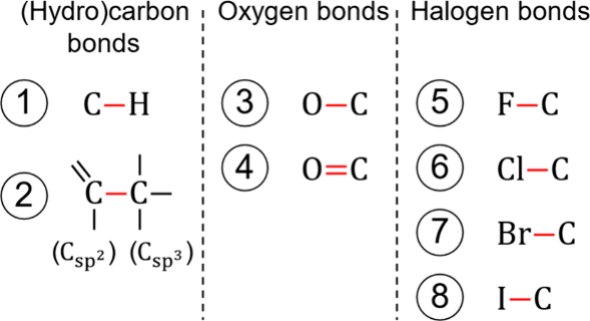
Defined bonds for the Vector-GC, categorized into (hydro)carbon (left), oxygen (center), and halogen (right) bonds. Letters represent atoms, connected by lines that represent bonds. The red lines indicate the defined bonds.

Next to groups, we need to define and parametrize bonds for the proposed dipole moment sum rule (cf., Section 2.2). We consider all bonds where an asymmetrical charge distribution is expected (Figure 3). In simple terms, asymmetrical charge distributions along a bond can be explained by a difference in electronegativity. Hence, bonds between atoms of different types should have nonzero dipole moment contributions. Additionally, a significant asymmetric charge distribution is also evident along bonds between differently hybridized carbon atoms.^36^

Based on this rationale, the hydrocarbon backbone for alkanes and alkenes has two bonds with dipole moment contributions: The carbon–hydrogen bond (C–H) and the bond between the sp^2^ and sp^3^ hybridized carbon atoms (C_sp^2^_–C_sp^3^_). However, we assume vanishing bond dipole contributions for these two bonds (μ_C–H_ = 0 and ) to ensure consistency for alkanes and alkenes with Sauer et al.^26^ (cf., adopted first-order carbon groups in Table 1). Sauer et al. modeled alkanes and alkenes as strictly nonpolar, which corresponds to the vanishing bond dipole moments for the hydrocarbon backbone.

To model oxygenated and halogenated substances, we consider bond dipole moment contributions for a single oxygen–carbon bond (O–C), a double oxygen–carbon bond (O=C), and for halogen−carbon bonds (F–C, Cl–C, Br–C, I–C).

In summary, we define 25 groups and eight bonds for oxygenated and halogenated substances. This work parametrizes 18 out of the 25 groups, while we adopt seven first-order carbon groups from Sauer et al.^26^ Moreover, we assume dipole moment contributions, *μ*_*β*_, of zero for the two hydrocarbon bonds, while parametrizing the contributions for the two oxygen–carbon and four halogen−carbon bonds.

### 3.2 Objective Function for the Parametrization

To yield optimal Vector-GC parameters for PCP-SAFT, we regress the group and bond contributions simultaneously against thermophysical property data. As an objective function to parametrize PCP-SAFT, the combination of vapor pressures and liquid densities has proven particular powerful.^40^ We employ a weighted least-squares sum of logarithmic deviations:
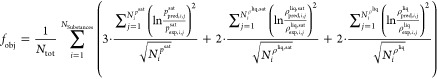
5Here, *N*_tot_ is the total number of data points for vapor pressures *p*^sat^, liquid densities ρ^liq^, and saturated liquid densities ρ^liq,sat^. Correspondingly, *N*_*i*_^*p*^sat^^, *N*_*i*_^ρ^liq^^, and *N*_*i*_^ρ^liq,sat^^ are the number of data points for substance *i*. *N*_Substances_ represents the number of considered substances. The employed experimental data is indicated by *p*_exp,*i*,*j*_^sat^, ρ_exp,*i,j*_^liq^, and ρ_exp,*i,j*_^liq,sat^ for substance *i* and state *j*, whereas the predictions obtained with the PCP-SAFT EoS are represented by *p*_pred,*i,j*_^sat^, ρ_pred,*i*,*j*_^liq^, and ρ_pred,*i*,*j*_^liq,sat^.

The employed objective function follows the weighting approach of Ramírez-Vélez et al.,^40^ who proposed regressing PCP-SAFT parameters against vapor pressure *p*^sat^ and saturated liquid density ρ^liq,sat^ data with weight factors of ω_*p*^sat^_ = 3 and ω_ρ^liq,sat^_ = 2. Since liquid density data is not available for all considered substances at saturation, we additionally consider liquid densities in the pure liquid phase ρ^liq^. To account for the uneven distribution of data points across substances and properties, we additionally weight the deviations of each substance and property according to the square root of the corresponding number of data points, as discussed by Rueben et al.^41^

We minimize the objective function by regressing the adjustable group and bond contributions as defined in Section 3.1. To this end, we define a lower bound of zero for the contributions of all first-order groups and all bonds. Since the second-order groups yield a correction to the contributions of the first-order groups, second-order group contributions are allowed to take negative values. A detailed overview of the employed initial values for the regression is provided in the Supporting Information (Table S1).

For validation purposes, we perform a leave-one-out cross-validation (LOO–CV): Here, we leave out not only a single data point but a single substance, i.e., performing the regression *N*_Substances_ times with *N*_Substances_-1 data sets. In each run, the data set of one substance is removed from the regression data set. To this end, we regard cis- and trans-isomers as one substance and remove the data of both isomers from the regression data set. The vapor pressures and liquid densities of the left-out substance are then predicted based on the regression to the data of the remaining *N*_Substances_-1 substances, or *N*_Substances_-2 substances in the presence of cis- and trans-isomers, respectively. Compared to a simple regression, the LOO–CV deviation yields a more accurate measure for the predictive capability of a model as it simulates the application of the model to substances that are not included in the regression data set. By this means possible overfitting to the regression data can be identified.

### 3.3 Property Data

Vapor pressure and liquid density data are taken from the Dortmund Data Bank (DDB, Version 2022),^42^ the ThermoML database,^43,44^ and the DIPPR 801 (May 2021) database.^45^ The data is curated as described in Esper et al.^46^ We regress group contributions separately for the two considered substance classes of oxygenated and halogenated substances. For this purpose, we create two separate data sets by extracting data from the databases for all aliphatic, noncyclic, noncharged, and nonassociating substances containing C, H, O and C, H, F, Br, Cl, I atoms, respectively. We further exclude data of substances with less than three and more than eight carbon atoms to restrict the parametrization to substances that are tangible with GC methods.

The defined filter steps finally yield 158 oxygenated and 95 halogenated substances. The data set for oxygenated substances comprises 24474 data points for vapor pressures, 21645 data points for subcooled liquid densities, and 445 data points for saturated liquid densities. For halogenated substances, we obtain 10392 vapor pressure, 33223 subcooled liquid density and 732 saturated liquid density data points. A detailed list of the considered substances is provided in the Supporting Information.

### 3.4 Computational Methods

To solve the defined minimization problem in Python, we employ the SciPy^47^ package (v1.11.4; solver *scipy.optimize.minimize*). For rapid computation of equilibrium properties with PCP-SAFT, the FeO_s_^48,49^ software package (v0.5.1) is used. Specifically, the PyTorch^50^ based implementation FeO_s_-torch^31,51^ is employed, leveraging reverse mode automatic differentiation.

### 3.5 Accessibility

The Vector-GC method can be easily employed through an openly available python package, predicting PCP-SAFT parameters directly from SMILES for the regressed substance classes. All regressed parameters are provided as JSON and CSV files. Moreover, the developed regression framework for group-contribution methods for PCP-SAFT is published open-source. The regression framework can be employed to regress the Vector-GC method to other substance classes and is also easily adaptable to other sum rules for PCP-SAFT.

## 4 Results and Discussion

We compare the performance of the Vector-GC method for vapor–liquid equilibrium and pure liquid density predictions resulting from the LOO–CV run to experimental data (cf., Section 3.2). To this end, we assess the method’s prediction performance for a substance *i* and a property ξ∈{*p*^sat^,ρ^liq,sat^,ρ^liq^} through the mean absolute percentage deviation (MAPD_*i*_^ξ^) between the LOO–CV predictions and the experimental data points:
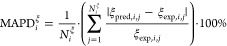
6

As an indicator for the predictive capabilities over a whole data set, we use the median of the substances’ percentage deviations.

To compare the Vector-GC with common GC methods, two further cases are considered: (1) assumption of μ = 0 for all substances (default approach of previous GC methods) and (2) the GC method of Sauer et al.^26^ for monofunctional oxygenated substances. For the μ = 0 method, we perform regressions and LOO–CV runs as described for the Vector-GC (cf., Section 3) but setting *μ*_*β*_ = 0 as constraint. The resulting group and bond dipole moment contributions from the Vector-GC and μ = 0 regressions are provided in the Supporting Information (Table S2).

In Section 4.1, we discuss vapor–liquid equilibrium (VLE) and pure liquid phase density predictions for the considered data sets of oxygenated and halogenated substances. Section 4.2 focuses on VLE predictions for cis–trans-isomers.

### 4.1 Prediction of Vapor–Liquid Equilibria

**Figure 4:**
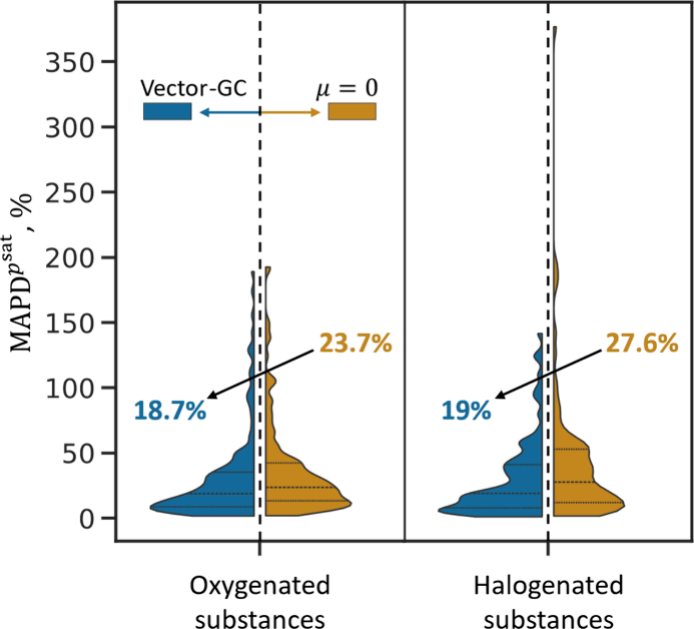
Distribution of mean average percentage deviations in vapor pressures (*MAPD*^*p*^*sat*^^) resulting from the predictions (LOO–CV) for oxygenated (left) and halogenated (right) substances. For each considered data set, the violin plot is split by method: Vector GC in blue and μ = 0 in orange. The horizontal black dashed lines inside the violin plots represent the quartiles in the *MAPD*^*p*^*sat*^^ distribution of the corresponding data set. The colored numbers correspond to the median values of each distribution.

The results in Figure 4 show the capability of the proposed Vector-GC method to predict vapor pressures. The prediction accuracy is substantially improved compared to the μ = 0 method that neglects the polar contributions for all substances. While the Vector-GC already decreases the median percentage deviations for oxygenated substances by 5 percentage points (Figure 4, left) from 23.7% to 18.7%, the median percentage deviations for halogenated substances is reduced by approximately 8 percentage points (Figure 4, right) from 27.6% to 19%. Moreover, the Vector-GC reduces the maximal percentage deviation in the data set of halogenated substances substantially from approximately 380% to approximately 140%, thus leading to more reliable predictions.

The Vector-GC method can be compared to the GC method of Sauer et al.^26^ for the subset of monofunctional oxygenated substances that are fragmentable with the Sauer et al. groups. For this subset containing 93 monofunctional oxygenated substances, the μ = 0 method yields a median percentage deviation of 21.7%, the Vector-GC yields a median value of 13.3%, and the Sauer et al. GC method results in a median of 10.4%. Hence, the Vector-GC yields larger deviations for monofunctional oxygenated substances compared to the Sauer et al. GC method. Note that Sauer et al. consider the dipole contribution of each of their six oxygen-containing groups as a freely adjustable parameter within the GC regression, while the Vector-GC considers only two bond contributions (μ_C–O_, μ_C–O_) and imposes physical constraints through to the employed 3D geometry estimate (cf., Section 2.2). This higher number of degrees of freedom of the Sauer et al. method could explain the lower deviation. However, the GC method of Sauer et al. is limited to substances with a single polar group, while the Vector-GC does not impose restrictions on the number of polar groups.

**Figure 5:**
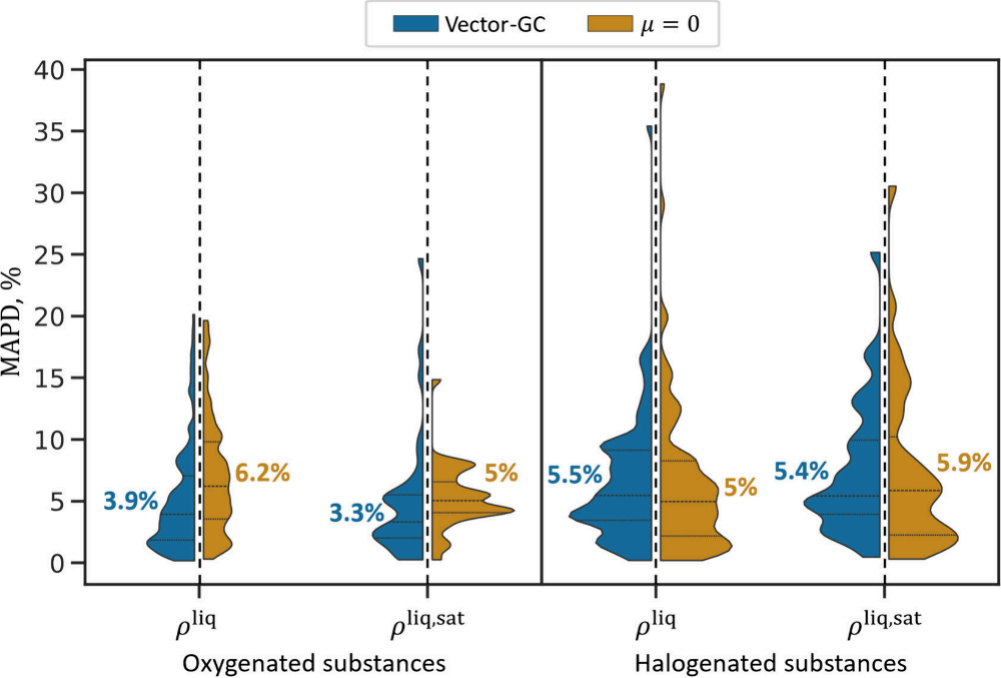
Distribution of mean average percentage deviations in liquid densities (*MAPD*^ρ^liq^^ and *MAPD*^ρ^liq,sat^^) resulting from the predictions (LOO–CV) for oxygenated (left) and halogenated (right) substances. For each considered data set, the violin plot is split by method: Vector GC in blue and μ = 0 in orange. The horizontal black dashed lines inside the violin plots represent the quartiles in the distributions of the corresponding data set. The colored numbers correspond to the median values of each distribution. Note that for saturated liquid densities, states above the predicted critical temperature cannot be predicted and, thus, the corresponding data points are excluded from the analysis. For the shown comparison between the different models, Vector-GC and μ = 0, we use the lower predicted critical temperature as cutoff.

In addition to vapor pressures, the objective function minimizes deviations in liquid densities (cf., Equation 5). In contrast to vapor pressures, the Vector-GC increases the overall prediction accuracy for liquid densities only slightly (Figure 5). For oxygenated substances, the Vector-GC decreases the median percentage deviation by approximately 2 percentage points for saturated and pure phase liquid densities (Figure 5, left). In the case of halogenated substances, the Vector-GC slightly increases the prediction accuracy for saturated liquid densities by 0.5 percentage points, while yielding a 0.5 percentage points higher deviation for pure phase liquid densities than the μ = 0 method (Figure 5, right).

For liquid densities, the comparison to the Sauer et al.^26^ GC method shows only slight differences between the methods. Saturated liquid densities of monofunctional oxygenated substances are predicted with median percentage deviations of 3.7%, 2.8%, and 4.7% by the Sauer et al. method, Vector-GC, and the μ = 0 method, respectively. For pure phase liquid densities, the Sauer et al. method yields a median percentage deviation of 3.3%, the Vector-GC results in a median of 3.4%, and the μ = 0 method yields a median percentage deviation of 5.9%.

**Figure 6:**
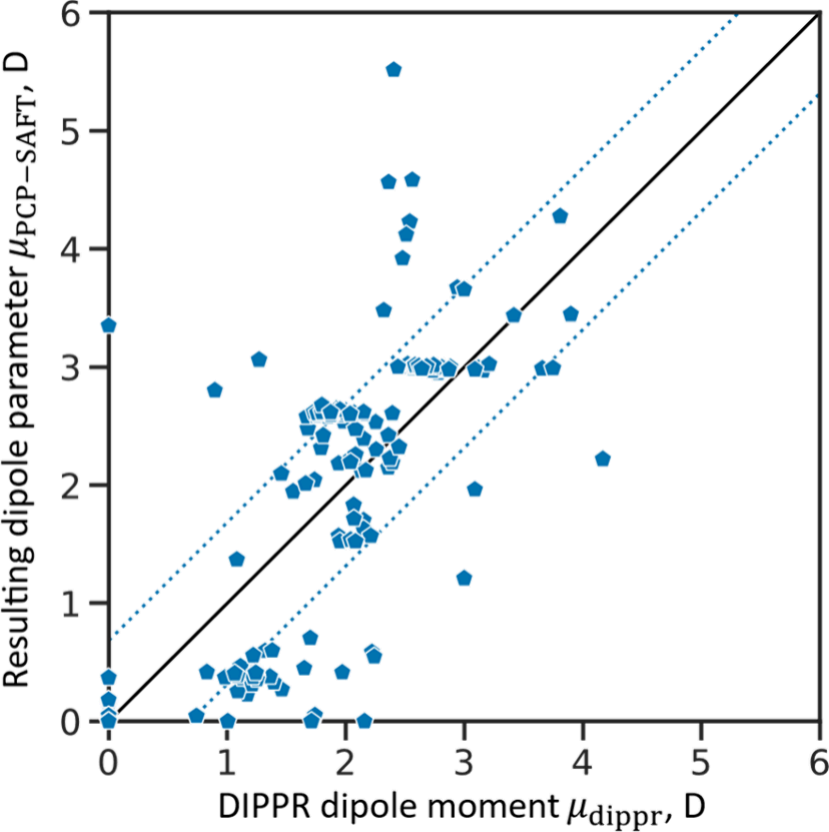
Parity plot for Vector-GC predicted PCP-SAFT dipole parameters against molecular dipole moment data from DIPPR^36^ that is available for the considered oxygenated and halogenated substances. In total, dipole moment data for 181 substances is plotted (130 oxygenated and 51 halogenated substances). The solid black line represents the angle bisector. The dashed blue lines indicate a deviation of 0.68 D, representing the mean absolute deviation.

The overall accuracy increase obtained by considering polar contributions is in line with previous studies,^8,13,15^ showing the relevance of the polar term in general. The Vector-GC method accurately and consistently incorporates the polar contributions into a GC method for PCP-SAFT. A comparison with experimental dipole moment data from the DIPPR database^45^ shows that the Vector-GC predicts PCP-SAFT’s dipole parameter physically sound (Figure 6). While the bond contributions are regressed to vapor pressure and liquid density data (cf., Section 3.2), the physical constraints imposed by the vector sum rule (cf., Section 2.2) ensures physically sound dipole parameters. The clear correlation between the resulting dipole parameters and the experimental dipole data is confirmed by a Pearson correlation coefficient of 0.7. The correlation to experimental dipole moment data can be slightly improved by regressing the bond contributions directly against the experimental data (an analysis is provided in the Supporting Information). However, regressing the bond contributions simultaneously with the other group contributions to vapor pressures and liquid densities ensures optimal parameters for use in PCP-SAFT. Additionally, the regression to thermodynamic data provides the flexibility to account for polarizability, while experimental dipole moment data is usually measured in the vacuum.

### 4.2 Cis- and Trans-Isomers

A specific feature of the Vector-GC method is enabling differentiation between cis- and trans-isomers. Generally, (first-order) GC methods are fundamentally limited by their inability to differentiate between isomers because a substance is solely represented by the occurrences of groups. Introducing second-order groups into a GC method can differentiate between additional structural isomers. However, cis- and trans-isomerism is a property of a substance’s 3D structure, which is generally not accessible even in higher-order GC methods. The dipole moment is the essential property that fundamentally differentiates cis- from trans-isomers. The proposed Vector-GC method accounts for the substance’s 3D structure and predicts dipole moments on a physically sound basis, thus differentiating between cis- and trans-isomers.

**Figure 7:**
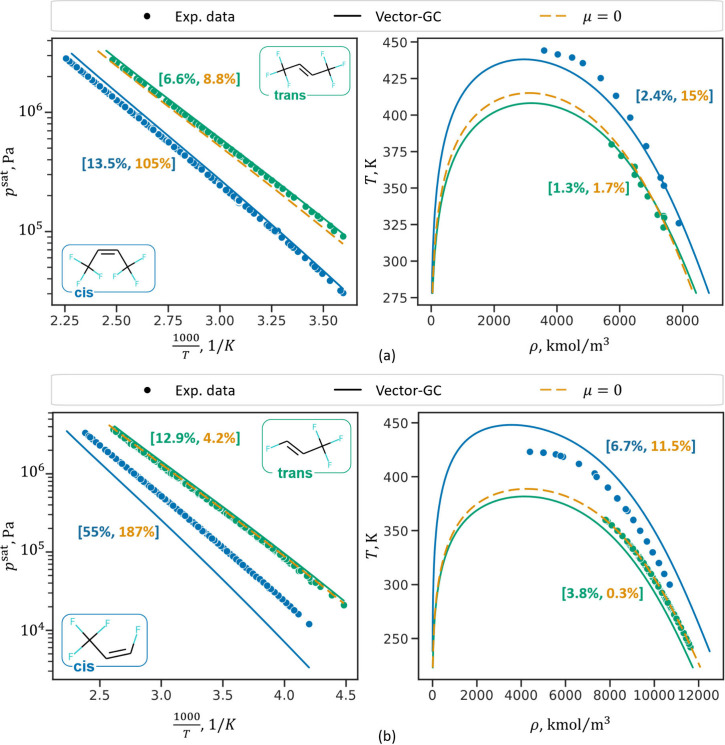
Prediction results (LOO–CV) for vapor pressures and saturated VLE densities for cis- and trans-hexafluoro-2-butene (a) and cis- and trans-tetrafluoroprop-1-ene (b). The blue and green dots represent experimental data for cis- and trans-isomers, respectively. The blue and green lines represent the Vector GC prediction for the cis- and trans-isomers, and the dashed orange line represents the prediction of the μ = 0 method, which is identical for both cis- and trans-isomers. The blue and green square brackets show the percentage deviations, [*MAPD*_*Vector*-*GC*_^*p*^sat^^,*MAPD*_μ = 0_^*p*^sat^^ ], of the Vector-GC and μ = 0 method for the cis- and trans-isomers, respectively.

Figure 7 shows results for vapor pressure and saturated liquid density predictions for the isomers of hexafluoro-2-butene (a) and tetrafluoroprop-1-ene (b). The example of hexafluoro-2-butene is of particular interest: Due to the significant difference in vapor pressures, only the cis-isomer is commonly employed as refrigerant (R-1336mzz(Z)). Differentiation between these isomers is therefore essential for property prediction within process simulators or molecular- and process design studies. The results in Figure 7 (a) show that the Vector-GC predicts vapor pressures and saturated liquid densities well for both isomers, while the μ = 0 method cannot differentiate between cis- and trans-isomers. Specifically, the μ = 0 method happens to predict the vapor pressures of the trans-isomer with high accuracy but leads to unreliable predictions for the cis-isomer. Similarly, the predictions for tetrafluoroprop-1-ene (Figure 7 (b)) show that the Vector-GC successfully differentiates between the cis- and trans-isomer, whereas the μ = 0 method only yields a single prediction. Here, the μ = 0 method results in lower deviations for the trans-isomer compared to the Vector-GC but again yields unreliable predictions for the cis-isomer. In contrast, the Vector-GC predicts the correct trend between the isomers, thus leading to substantially lower deviations for the cis-isomer.

**Figure 8:**
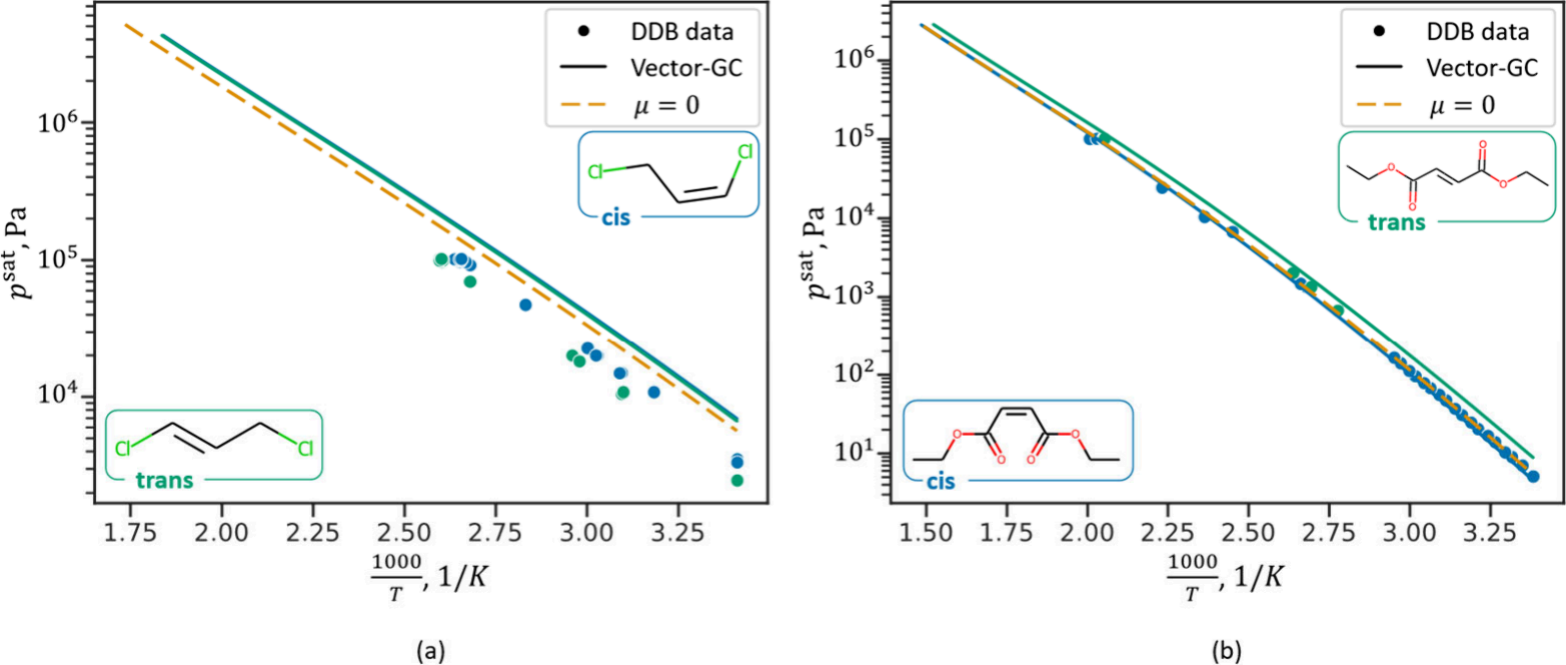
Prediction results (LOO–CV) for cis- and trans-1,3-dichloro-1-propene (a) and cis- and trans-diethyl-but-2-enedioate (b). The blue and green dots represent experimental data for cis- and trans isomers, respectively. The blue and green lines represent the Vector GC prediction. The dashed orange line represents the prediction of the μ = 0 method, which is identical for both cis- and trans-isomers. Note that the data of trans-diethyl-but-2-enedioate was not included in the regression data set as not enough temperature bins are occupied in the vapor pressure data (cf., filter steps described in^40^).

Figure 8 shows examples for cis- and trans-isomers with similar properties. In the case of 1,3-dichloro-1-propene (Figure 8 (a)), the Vector-GC predicts similar dipole moments for the cis- and trans-isomer: μ_pred_^cis^ = 2.32 (μ_dipper_^cis^ = 1.79) and μ_pred_^trans^ = 2.42 (μ_dippr_^trans^ = 1.81). The Vector-GC therefore captures the underlying physics, leading to the correct prediction of similar vapor pressures of cis- and trans-dichloropropene. For diethyl-but-2-enedioate (Figure 8 (b)), the Vector-GC predicts significantly different dipole moments for the isomers: μ_pred_^cis^=4.58 (μ_dippr_^cis^ = 2.56) and μ_pred_^trans^ = 0.03 (μ_dippr_^trans^ = none). The trend in the predicted dipole moments is physically reasonable. However, due to the substance’s size, the polar contribution does not have the same influence as for the discussed smaller substances. Hence, the physical sound basis of PCP-SAFT correctly predicts similar vapor pressure in this case.

## 5 Conclusion

A Vector-GC method is proposed to predict PCP-SAFT parameters of dipolar substances, incorporating polar contributions without limitation on the allowed number of polar groups. The Vector-GC method predicts molecular dipole moments based on a vector sum of bond dipole moment contributions. The Vector-GC method accounts for the molecular 3D structure by employing a force field estimate in the fragmentation step. Considering the 3D structure overcomes the nonadditivity problem of dipole moments caused by molecular symmetries. The Vector-GC method can be easily employed through an openly available python package, predicting PCP-SAFT parameters directly from SMILES for the regressed substance classes.

To regress the Vector-GC method and evaluate its performance, we use a data set of vapor pressures and liquid densities for nonassociating, oxygenated substances (158) and halogenated substances (95). All regressed parameters are provided as JSON and CSV files. Moreover, the developed regression framework is openly available and can be employed to regress the Vector-GC method to further substance classes.

We demonstrate that the Vector-GC accurately and consistently incorporates the dipolar contributions into a GC method for PCP-SAFT. In comparison to the common approach of neglecting polar contributions (μ = 0), the Vector-GC reduces the median values of the mean absolute percentage deviations by approximately 5 percentage points for oxygenated substances and 8 percentage points for halogenated substances. The final median percentage deviations are approximately 19% for both oxygenated and halogenated substances. Furthermore, we show that the Vector-GC improves the median prediction accuracy for liquid densities of oxygenated substances by approximately 2 percentage points.

The dipole moments predicted by the Vector-GC method are physically sound (mean absolute deviation of 0.68 D) and correlate well with experimental data from the DIPPR database (Pearson correlation coefficient of 0.7). We expect this physically sound dipole moment prediction to be particularly relevant for predicting mixture properties of dipolar substances with PCP-SAFT. Specifically, we expect an improved prediction for nonpolar/dipolar and dipolar/dipolar mixtures, while mixtures of dipolar and associating substances have been shown to rely on binary interaction parameters.^31^

Finally, we demonstrate that the Vector-GC method enables differentiation between cis- and trans-isomers. The Vector-GC method predicts the correct trends for all pairs of cis- and trans-isomers included in the studied data sets and, thereby, substantially increases the applicability domain and prediction capabilities of GC methods.

The Vector-GC method enhances predictive thermodynamics through group-contribution methods. Their applicability domain and prediction capabilities are expanded, in particular, for dipolar substances and cis- and trans-isomers. Due to the leave-one-out cross-validation conducted in our study, we expect good transferability to similar substances outside the data sets considered. The proposed Vector-GC method is not restricted to PCP-SAFT but can be adapted to other SAFT models that consider the dipole moment as model parameter.

## Data Availability

Experimental property data underlying this study cannot be shared publicly as they were provided under license by DECHEMA, DDBST GmbH, and the Design Institute for Physical Properties (DIPPR). The Vector-GC method and the developed regression framework are published open-source on GitLab, at https://gitlab.ethz.ch/epse/molecular-design-public/vector-gc, under the MIT license. Specifically, the GitLab repository contains code to run the Vector-GC model based on a SMILES input, JSON and CSV files with the regressed parameters, source code of the developed regression framework utilizing automatic differentiation via FeO_s_-torch^31,51^ and a minimal working example for the regression.

## Supporting Information Available

Details on initial values for the regressions, the resulting group and bond contributions, and an analysis on the direct regression of bond contributions to experimental dipole moment data (PDF)
Resulting mean deviations (regression and LOO–CV) in vapor pressures and liquid densities for all considered substances and the resulting PCP-SAFT parameters for all considered substances (XLSX)

## Nomenclature Variables

Variable: Description, Unit
*n*: Occurrence of a group in a substance, –
*m*: PCP-SAFT parameter (chain length), –
σ: PCP-SAFT parameter (chain size), Å
ε: PCP-SAFT parameter (depth of potential well), J
μ: PCP-SAFT parameter (dipole moment), *D*
ε_AB_: PCP-SAFT parameter (association energy), J
κ_AB_: PCP-SAFT parameter (association volume), –
*N*: Number of data points
*p*: Pressure, Pa
ρ: Molar density, mol/m^3^
MAPD: Mean absolute percentage deviation,%

## Greek letters

Letter: Description
α: Group
β: Bond
ξ: Property
δ: Data set

## Acronyms and Abbreviations

Acronym: Description
SAFT: Statistical associating fluid theory
PC: Perturbed-chain
PPC: Polar perturbed-chain
PCP: Perturbed-chain polar
EoS: Equation of state
GC: Group-contribution
DDB: Dortmund Data Bank
DIPPR: Design Institute for Physical Properties
LOO–CV: Leave-one-out cross-validation

## Sub- and superscripts

Subscript: Description
*i*: Substance
*j*: State defined by temperature and/or pressure
pred: Prediction
exp: Experimental data
tot: Total
Vector-GC: Vector-GC method
μ = 0: Benchmark case of neglecting the dipole term

## Superscript Description

sat: Saturated
liq: Liquid
pure: Pure phase
cis: Isomer with cis configuration
trans: Isomer with trans configuration
