# Supplementary material for: Modeling Dipolar Molecules with PCP-SAFT: A Vector Group-Contribution Method
Source: ACS Omega. 2024 Sep 5;9(37):38809–19. doi: 10.1021/acsomega.4c04867 (PMC11411541; doi:10.1021/acsomega.4c04867)
Supplement: Supplementary file 1 — ao4c04867_si_001.pdf [file ao4c04867_si_001.pdf]

Supporting Information:
Modeling dipolar molecules with PCP-SAFT: A Vector Group-Contribution method

Carl Hemprich,[†] Philipp Rehner,[†] Timm Esper,[‡] Joachim Gross,[‡] Dennis Roskosch,[†] André Bardow[*,†]

†Energy and Process Systems Engineering, Department of Mechanical and Process Engineering, ETH Zurich, Tannenstrasse 3, 8092 Zurich, Switzerland

‡Institute of Thermodynamics and Thermal Process Engineering, University of Stuttgart, Stuttgart 70569, Germany

E-mail: abardow@ethz.ch

Contents

***Supporting Information:* Modeling dipolar molecules with PCP-SAFT: A Vector Group-Contribution method** 1

Initial values for the regression 2

Resulting group and bond contributions 3

Direct regression to experimental dipole moment data 4

References 6

Initial values for the regression

Table S 1 Initial values employed for the regression of both the Vector-GC and the μ=0 method. Initial values for oxygenated groups are chosen based on the corresponding group contributions of Sauer et al. [23].

**Group α**	m_α, –	σ_α, Å	ε_α/k, K
$-CH=O$	1.58	2.8	243.0
$>C=O$	1.22	2.81	249.0
$-O-$	1.7	3.1	196.0
$-O-CH=O$	1.76	2.9	230.0
$-O-(C=O)-$	1.51	2.82	223.0
$-F$	0.3	3.5	350.0
$-Cl$	0.4	3.5	350.0
$-Br$	0.5	3.5	350.0
$-I$	0.6	3.5	350.0
$SO: (-CH_2-)_{Halo}$	0.0	3.0	0.0
$SO: (>CH-)_{Halo,middle}$	0.0	3.0	0.0
$SO: (>CH-)_{Halo,end}$	0.0	3.0	0.0
$SO: (>C<)_{Halo,branch}$	0.0	3.0	0.0
$SO: (>C<)_{Halo,middle}$	1.0	3.0	100.0
$SO: (>C<)_{Halo,end}$	1.0	3.0	100.0
$SO: (=CH-)_{Halo}$	0.0	3.0	0.0
$SO: (=C<)_{Halo,middle}$	0.0	3.0	0.0
$SO: (=C<)_{Halo,end}$	0.0	3.0	0.0
**Bond β**	**μ_β in D**		
$O-C$	0.7		
$O=C$	2.4		
$F-C$	1.5		

$Cl-C$	1.5
$Br-C$	1.5
$I-C$	1.5

Resulting group and bond contributions

Table S 2 Resulting group parameters for Vector-GC and the μ=0 method for regression to vapor pressure and liquid density data.

	**Vector-GC**			$\mu = 0$		
**Group α**	$m_\alpha, -$	σ_α, Å	ε_α/k, K	$m_\alpha, -$	σ_α, Å	ε_α/k, K
$-CH=O$	1.197	3.293	276.07	1.623	3.092	253.63
$>C=O$	0.916	3.017	304.44	1.348	2.967	260.26
$-O-$	0.762	2.616	189.53	1.421	2.045	147.87
$-O-CH=O$	1.732	2.867	236.18	1.779	2.908	245.88
$-O-(C=O)-$	1.693	2.636	225.81	1.653	2.900	236.06
$-F$	0.409	2.146	214.32	0.272	3.019	267.11
$-Cl$	0.243	4.593	645.92	0.490	3.787	362.55
$-Br$	0.708	3.583	369.46	1.066	3.111	277.79
$-I$	0.856	3.767	393.98	0.898	4.032	364.74
$SO: (-CH_2-)_{Halo}$	-0.106	1.150	-191.33	-0.185	2.140	-144.60
$SO: (>CH-)_{Halo,middle}$	0.450	3.032	96.210	-0.055	2.402	-30.903
$SO: (>CH-)_{Halo,end}$	1.920	1.926	96.698	-0.086	1.464	-53.328
$SO: (>C<)_{Halo,branch}$	0.022	3.519	-80.758	0.019	2.978	15.460
$SO: (>C<)_{Halo,middle}$	0.348	4.221	-7.5328	0.615	2.871	40.239
$SO: (>C<)_{Halo,end}$	0.820	3.320	21.960	1.298	2.733	58.776
$SO: (=CH-)_{Halo}$	0.109	5.070	-109.84	0.023	3.133	17.371
$SO: (=C<)_{Halo,middle}$	0.084	-0.104	-343.88	0.179	0.778	-93.067
$SO: (=C<)_{Halo,end}$	0.353	-0.736	-173.92	0.553	0.785	-89.350

**Bond β**	**μ_β in D**	
$C_{sp2} - C_{sp3}$	0.0 (by assumption)	
$C - H$	0.0 (by assumption)	
$O - C$	0.345	
$O = C$	2.983	
$F - C$	2.071	
$Cl - C$	1.956	
$Br - C$	1.936	
$I - C$	1.903	

Direct regression to experimental dipole moment data

In the main paper, we regress the bond contributions of the proposed dipole moment sum rule simultaneously with the other group contributions to thermodynamic data. In addition to this approach, we here investigate the minimally achievable deviation between experimental dipole moment data and the proposed dipole moment sum rule, i.e., the sum rule's model error. For this purpose, we regress the defined adjustable bond dipole contributions ($\mu_{\mathrm{F-C}}, \mu_{\mathrm{Cl-C}}, \mu_{\mathrm{Br-C}}, \mu_{\mathrm{I-C}}, \mu_{\mathrm{I-C}}, \mu_{\mathrm{O-C}}$, and $\mu_{\mathrm{O=C}}$) against the molecular dipole moment data from the DIPPR [1] database. The DIPPR database contains molecular dipole moment data for 181 of the 253 substances from the defined data sets of oxygenated and halogenated substances (cf., Section 3 in main paper).

To this end, we minimize the least square sum of the deviation between predicted and experimental molecular dipole moment:

$$f_{obj} = \frac{1}{N_\mu} \sum_{i=1}^{N_\mu} \left(\mu_{\mathrm{dippr},i} - \mu_{\mathrm{pred},i}\right)^2. \qquad (1)$$

Here, $N_\mu = 181$ is the total number of available dipole moments in the DIPPR database for the considered case study, $\mu_{\mathrm{dippr},i}$ denotes the DIPPR dipole moment of substance i, and $\mu_{\mathrm{pred},i}$

represents the predicted dipole moment, determined by Equation (4) of the main paper. We define lower bounds for the considered bond dipole contributions such that $\mu_\beta \geq 0$ holds for all considered bonds β. We perform a leave-one-out cross-validation (LOO-CV).

A comparison to the PCP-SAFT dipole parameters predicted with the Vector-GC shows that regressing the bond contributions directly to experimental dipole moment data yields a lower mean absolute deviation (Figure S 1) and a slightly stronger correlation (Pearson correlation coefficient of 0.7 for Vector-GC, 0.73 for the regression to experimental data, and 0.71 for the LOO-CV prediction). This difference is expected due to the different objective functions chosen. Evidently, the Vector-GC uses the degrees of freedom of the sum rule to obtain dipole moments that are optimal for PCP-SAFT. PCP-SAFT expects effective dipole moments in the fluid phase [2], while the DIPPR database mainly contains dipole moments measured in vacuum. Hence, it is reasonable that the Vector-GC yields slightly higher dipole moments compared to regressing the dipole moments directly to the DIPPR data.

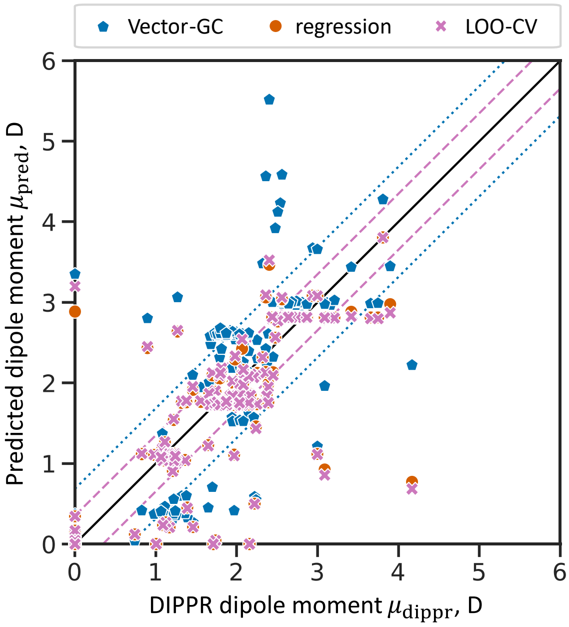

Figure S 1 Parity plot for predicted molecular dipole moments against molecular dipole moment data from DIPPR [39]. Blue pentagons represent dipole moments resulting from the Vector-GC method (LOO-CV), red dots represent the regression result against the DIPPR data and pink crosses represent the LOO-CV predictions. The solid black line represents the angle bisector. The dashed pink lines indicate a deviation of 0.35 D, which is the mean absolute deviation of the LOO-CV. The dotted blue lines indicate a deviation of 0.68 D, which is the mean absolute deviation resulting from the Vector-GC.

In addition, the comparison between the LOO-CV prediction and regression results shows only minor differences: The Pearson correlation coefficient is slightly higher for the regression results (LOO-CV:

0.71, regression: 0.73) and the mean absolute deviation is slightly lower (LOO-CV: 0.35 D, regression: 0.34 D). The strong similarity between LOO-CV and regression results show that the sum rule is robust and does not tend to overfitting for the considered data set.

The optimal bond dipole moments resulting from the regression to DIPPR data and from the Vector-GC are given in Table S 3.

Table S 3 Resulting bond dipole moments for regression to dipole moment data from the DIPPR database, compared to bond dipole moments resulting from the Vector-GC.

Bond β	Bond dipole moment regressed to DIPPR data, $\mu_\beta^{\mathrm{dippr-regression}}$, D	Bond dipole moment resulting from the Vector-GC, D
$\mathrm{C_{sp2} - C_{sp3}}$	0.0 (by assumption)	0.0 (by assumption)
$\mathrm{C - H}$	0.0 (by assumption)	0.0 (by assumption)
$\mathrm{O - C}$	0.997	0.345
$\mathrm{O = C}$	2.812	2.983
$\mathrm{F - C}$	1.917	2.071
$\mathrm{Cl - C}$	1.86	1.956
$\mathrm{Br - C}$	2.092	1.936
$\mathrm{I - C}$	2.002	1.903

References

[1] W.V. Wilding, T.A. Knotts, N.F. Giles, R.L. Rowley, DIPPR Data Compilation of Pure Chemical Properties, (2020).

[2] J. Gross, J. Vrabec, An equation-of-state contribution for polar components: Dipolar molecules, AIChE Journal 52 (2006) 1194–1204. https://doi.org/10.1002/aic.10683.
